# Development of a Web-Based School Support System Within the AVATAR Project for Psychosocial Well-being in Adolescents: Pilot Feasibility Study

**DOI:** 10.2196/24840

**Published:** 2021-12-02

**Authors:** Francesca Mastorci, Paolo Piaggi, Gabriele Trivellini, Cristina Doveri, Anselmo Casu, Luca Bastiani, Irene Marinaro, Cristina Vassalle, Alessandro Pingitore

**Affiliations:** 1 Clinical Physiology Institute Consiglio Nazionale delle Ricerche Area della Ricerca di Pisa (CNR) Pisa Italy; 2 Department of Information Engineering University of Pisa Pisa Italy; 3 Fondazione Toscana Gabriele Monasterio Pisa Italy

**Keywords:** adolescent, well-being management, schools, web tool, health promotion

## Abstract

**Background:**

Health and well-being promotions are key points of educational programs for adolescents within schools. There are several health education programs mainly based on lifestyle habit changes; however, social and emotional dimensions should be considered within these educational strategies.

**Objective:**

This study aimed to (1) develop a new web-based school support system to assess and analyze individual, classroom, and scholastic institute data on lifestyle habits, social context, emotional status, and scholastic performance; (2) create a web tool for managing the well-being of adolescents through a dynamic and personalized interface that provides immediate feedback that allows the school to monitor progress; and (3) evaluate, in a pilot study, the feasibility of this web-based school support system in order to build health programs that are specific to the needs of the studied population.

**Methods:**

The AVATAR (a new purpose for the promotion and evaluation of health and well-being among healthy teenagers) method consists of integrating the information coming from different questionnaires. In particular, to allow planning didactic and educational actions based on the results obtained, the AVATAR approach allows subdivision of the results of the different observed variables and the 4 components into the following 3 percentile categories: modify, improve, and maintain. The AVATAR web platform was designed to collect data on lifestyle, emotional status, and social context from junior high schools in terms of the fundamental aspects of adolescent daily life, with free use by the scholastic community (scholars, teachers, and parents). In this pilot/feasibility study, data from 331 students were acquired between 2018 and 2019 at the beginning of the scholastic year (pre) and at the end following the school-based program (post).

**Results:**

Preliminary results showed that after school planning and specific program implementation, defined after AVATAR feedback, students reported better well-being perception characterized by higher perception in psychological well-being (*P*=.001), mood (*P*=.001), self-perception (*P*=.006), and autonomy (*P*=.001), and an increase in the perception of financial resources (*P*=.001), which helped in developing healthy lifestyle habits (*P*=.007). In the social context assessment, students reported stronger relationships with family (*P*=.02) and peers (*P*=.001), and a lower perception of bullying (*P*=.001).

**Conclusions:**

The AVATAR web-based platform is a feasible and flexible tool for the health and well-being management of adolescents from epidemiological, preventive, and educational points of view. In particular, it can be used to (1) promote information campaigns aimed at modifying risk behaviors in the student population, (2) sensitize students and put them at the center of their growth path, (3) inform institutions about the health and well-being of the school population, (4) ensure health programs are acceptable and feasible to users before launching on a large scale, and (5) improve the relationship of users (school) and educational agencies with research groups.

## Introduction

Health and well-being promotions are key points of educational programs for adolescents within schools. Schools allow obtaining a large number of adolescents from different family social backgrounds and represent a more naturalistic and interactive environment to induce positive effects on health from social (eg, family, peers, and teachers) and cultural perspectives [1,2]. There are several health education programs, which are mainly based on lifestyle changes (in particular, diet and exercise), that significantly reduce the incidences of obesity and metabolic syndrome [3]. However, considering that different factors have impacts on health and well-being in adolescents, including those in the social, emotional, and mental context, these health components should be implemented within health education programs [4]. In this regard, the KIDSCREEN questionnaire is a standardized tool to assess the quality of life and well-being of adolescents, which has been validated in several European countries and includes items of different areas impacting health in adolescents, that can help to identify critical areas for health education program interventions [5]. In this way, health education programs can be more focused on the needs of adolescents through the identification of the items to improve and those to potentiate. In the perspective of an integrated and multidisciplinary framework of health intervention, our group developed an integrated and personalized index of well-being built on the integration of the weights of items belonging to the dimensions of lifestyle, social context, emotional status, and mental skills [6]. This index has been built for a single adolescent, where health interventions can be oriented in a personalized approach. However, in a school context, health programs need to be oriented to the class and institute community, taking into account the health profile of each student. This pushed us to develop a tool in which the data of students on the abovementioned dimensions were provided as classroom data and institution data.

This study aimed to examine the potential impact of the development of a web-based school support system to acquire, archive, and analyze online data about lifestyle habits, social context, emotional status, and scholastic performance in order to provide immediate feedback of the results to the school. The goal of this pilot study was to evaluate the feasibility of this web-based tool to monitor longitudinally the effects of health programs adopted in relation to questionnaire results.

## Methods

### Overview

In this pilot/feasibility study, first, we developed a web-based school support system to acquire, archive, and analyze in real time data about lifestyle habits, social context, emotional status, and scholastic performance among students. Second, we reported the methods and results of the feasibility study. All procedures performed in this study were in accordance with the ethical standards of the institutional and/or national research committee and with the 1964 Helsinki declaration and its later amendments or comparable ethical standards. The protocol was approved by the regional ethics committee (166/2018). In addition, the pilot/feasibility study was approved by the internal ethics committee of each participating school, in accordance with Italian law. All parents or legal guardians gave informed consent and authorized researchers to use the data in accordance with Italian law.

### General Aspects of the AVATAR Web-Based Tool

The web-based platform was designed to collect data from junior high schools participating in the AVATAR (a new purpose for the promotion and evaluation of health and well-being among healthy teenagers) project on the fundamental aspects of adolescent daily life, with free use without a commercial license by the scholastic community (scholars, teachers, and parents). In particular, variables on health and well-being that encompassed different dimensions of health, including lifestyle, emotional status, and social context, were monitored. The platform contained questionnaires; training documents; and reports accessible to schools, teachers, parents, and people active in the field of education and prevention. This allowed us to (1) define and understand the needs of the population (adolescence) for interventions or public health programs, (2) evaluate if the proposed programs are acceptable and feasible for users before launch through a pilot study, and (3) improve the relationship between users (at school) and research groups.

### Data Collection

Data from students at different times during the scholastic year (usually at the beginning and at the end of the scholastic year) were collected using the AVATAR web tool [7]. A sociodemographic data record was used to acquire information about gender, age, schooling, family structure, and BMI, according to World Health Organization age groups [8]. The Italian version of KIDSCREEN-52 was used to assess health-related quality of life [9,10]. The KIDSCREEN is a self-report questionnaire designed to assess health-related quality of life, with the aim to monitor and measure personal experiences in children and adolescents about their perceptions of health status and well-being. The questionnaire, which describes physical, psychological, mental, social, and functional aspects of well-being, consists of 52 items grouped into 10 dimensions [9,10]. The KIDSCREEN questionnaire has been psychometrically tested using data obtained in a multicenter European study that included a sample of 22,827 children recruited in 13 countries [11]. Dietary habits were evaluated using the Mediterranean Diet Quality Index for children and adolescents (KIDMED) [12]. The KIDMED index is based on principles sustaining Mediterranean dietary patterns, as well as those that undermine it. The index ranges from 0 to 12, and consists of a self-administered 16-question test. Physical activity levels were assessed using the Physical Activity Questionnaire for Older Children (PAQ-C). The questionnaire provides a general measure of physical activity for those aged 8 to 20 years. The PAQ-C is a self-administrated questionnaire consisting of 9 items rated on a 5-point scale. A higher score indicates more active children/adolescents [13].

The perception of school engagement was estimated through questions concerning scholastic achievements in language and literature, language acquisition, and science.

### Health-Related Quality of Life Components

In the AVATAR platform, the following 4 components of health-related well-being have been considered: lifestyle habits, emotional status, social context, and mental skills.

From our previous experimental evidence, the *lifestyle habits* component was hypothesized to cause changes in physical well-being, autonomy, financial resources, diet, and physical activity, while the *social context* component was hypothesized to cause changes in parent relations, peers, school environment, and bullying (analogous schemas were adopted for the social context and mental components).

Conceptually, the AVATAR methodology is based on a multidimensional construct, covering the physical, emotional, mental, and social components of well-being as perceived by adolescents [6,7,14,15]. The indicators belonging to the 4 components have been selected according to the analysis of pre-existing literature involving adolescents’ health and well-being [16-18]. Table 1 presents the individual variables (observed variables) for each component.

**Table 1:** Variables (observed variables) for each component.

Component	Well-being dimensions (observed variables)
Lifestyle habits	Physical well-being Autonomy Financial resources Diet Physical activity
Social context	Parent relations Peers School environment Bullying
Emotional status	Psychological well-being Mood Self-perception Emotion

### Data Management

The AVATAR method involves integrating the information coming from the different questionnaires. In particular, to allow planning didactic and educational actions based on the results obtained, the AVATAR approach allows subdivision of the results of the different observed variables and the 4 components into the following 3 percentile categories: modify, improve, and maintain.

To obtain this representation of the data, for each investigated area of the questionnaire, the cutoffs for adolescent-reported dimension and total scores were defined using the 10th and 90th percentiles, based on the sample distribution into 3 categories. Scoring in the ≤10th percentile was used to classify the proportion of adolescents having poor quality in each investigated area (orange; modify), scoring in the ≥90th percentile was used to classify the proportion of adolescents having high quality (blue; improve), and scoring between the 10th and 90th percentiles was used for intermediate values (green; maintain) [19,20].

**Figure 1:**
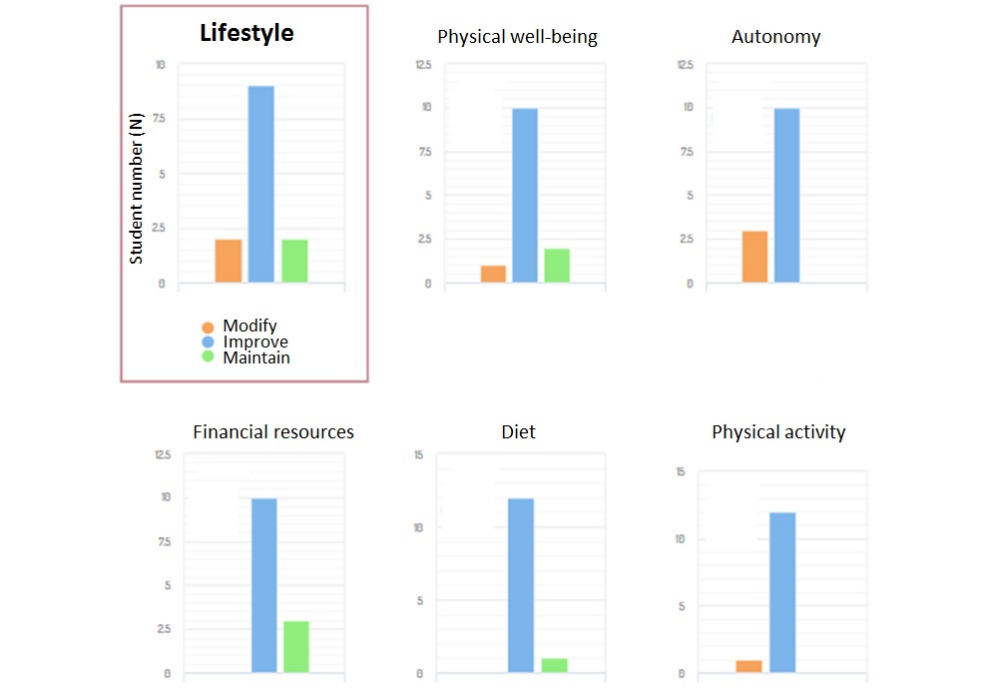
Example of data view. Results are expressed as being merged and integrated into a component (eg, lifestyle) or single variables of the entire class.

**Figure 2:**
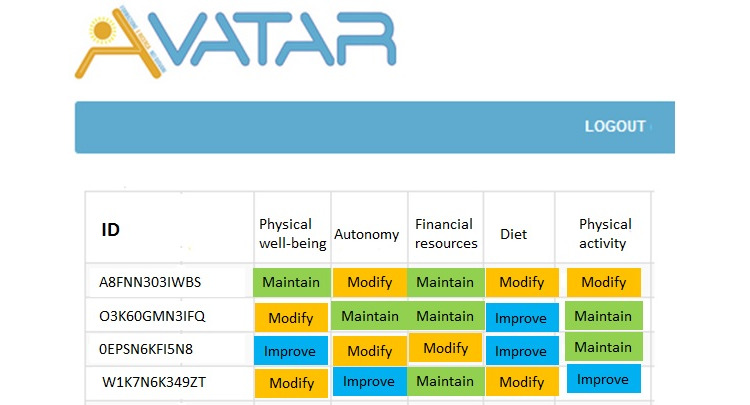
Details of the individual variables of a component (eg, lifestyle) for each student as shown on the platform classified as modify, improve, and maintain.

The teacher can view the data expressed as a single variable or merged and integrated into the 4 components of the entire class and the institution (Figure 1), up to the individual pupil, identified with an ID (Figure 2).

### Methods of the Pilot/Feasibility Study

In order to evaluate the feasibility of the AVATAR web-based school support system, a pilot study, as a part of the AVATAR project, was performed. Data collection was conducted between 2018 and 2019 from 1 of 10 junior high schools at the beginning (pre) and at the end (post) of the school year. In total, 331 students (172 female students, 52%; 159 male students, 48%; mean age 12.5 years, SD 1 year) were included. Adolescent students were enrolled according to the following inclusion criteria: age 10 to 14 years, absence of neuropsychiatric or other diseases, informed consent signed, and completion of the questionnaires proposed. In every school class, all adolescents filled out the questionnaires, and those who were not eligible were excluded from the study retrospectively. The questionnaires, which have been previously described, were filled at the beginning and end of the school year.

Participants were previously instructed on how to fill out the questionnaires and how to conduct the tests. All tests were conducted during participants’ computer lessons in school. No incentive was provided to adolescents or parents. A research assistant was available to provide information and technical support to complete the questionnaires.

### School-Based Program Built on the AVATAR Platform

With the results obtained by the AVATAR platform in the first AVATAR administration (at the beginning of the school year), school planning and specific program implementation were aimed to support the development of students’ emotional-relational skills and competences. In particular, the project and didactic actions were oriented on the basis of the identified needs and aimed to (1) strengthen the self-efficacy of students, as well as their social skills, to increase personal responsibility in relationships for developing autonomy (empathy, self-regulation, and self-efficacy) and (2) enhance citizenship skills (theatre, environment, art, counselling, nutrition, sports, breathing, relaxation, etc) with a view of prevention, which was integrated with curricular planning. For these reasons and for didactic/educational aims, the pilot study addressed the following 3 AVATAR areas: lifestyle habits, social context, and emotional status.

### Statistical Analysis

Statistical data analyses were performed using SPSS (version 22.0; IBM Corp). Data are presented as mean (SD) or as mean with 95% CI. Alpha was set at .05, and 2-sided *P* values have been reported. Changes in health-related quality of life (baseline vs post) were analyzed using the Student paired *t* test. The McNemar-Bowker test was used to evaluate changes in the proportion of subjects belonging to each tertile of health-related quality of life variables after the intervention.

## Results

### Effects of the School-Based Program on Health-Related Quality of Life and Lifestyle Habits

Descriptive data on health-related quality of life and lifestyle habits (diet and physical activity; from preprogram and postprogram) are presented in Table 2. Data on the KIDSCREEN-52 dimensions are calculated as mean T-scores according to the KIDSCREEN Group [9,10]. During the school year, following the program built on the results of the first administration, students showed a higher perception in psychological well-being (*P*=.001), mood (*P*=.001), self-perception (*P*=.006), and autonomy (*P*=.001), which was understood as the opportunity to create his/her social and leisure time. There was an increase in the well-being perception owing to the school-based program, as well as the perception of financial resources compared with the initial situation (*P*=.001). In the social context, students reported higher values in the relationship with their family (*P*=.02) and peers (*P*=.001), and exhibited a lower perception of bullying (*P*=.001). For lifestyle habits, after the personalized program, students developed higher adherence to the Mediterranean diet (*P*=.007) and higher physical activity levels (*P*=.001) compared with the previous condition.

**Table 2:** Questionnaire findings in the pilot study sample at the beginning (pre) and end (post) of the school year.

Variable			Pre score (n=331), mean (SD)		Post score (n=331), mean (SD)		*P* value^a^
**KIDSCREEN-52^b^**							
	Physical well-being	47.35 (SD 18.52)		47.61 (SD 22.05)		.85	
	Psychological well-being	44.50 (SD 15.86)		52.59 (SD 9.67)		.001	
	Mood/emotion	46.95 (SD 13.52)		50.71 (SD 10.76)		.001	
	Self-perception	51.45 (SD 15.8)		53.79 (SD 11.32)		.006	
	Autonomy	45.21 (SD 14.21)		50.96 (SD 10.85)		.001	
	Parent relationship	51.55 (SD 11.52)		53.08 (SD 10.27)		.02	
	Financial resources	47.66 (SD 13.44)		52.20 (SD 8.95)		.001	
	Peers	50.15 (SD 12.04)		53.06 (SD 11.01)		.001	
	School environment	50.34 (SD 10.43)		51.24 (SD 9.38)		.07	
	Social acceptance	46.02 (SD 15.22)		49.57 (SD 10.67)		.001	
KIDMED^c^			5.76 (SD 2.42)		6.13 (SD 2.45)		.007
PAQ-C^d^			2.57 (SD 0.67)		2.76 (SD 0.66)		.001

^a^*P* values were calculated using the Student paired *t* test.

^b^Data on the KIDSCREEN-52 dimensions were calculated as mean T-scores according to the KIDSCREEN Group.

^c^KIDMED: Mediterranean Diet Quality Index for children and adolescents.

^d^PAQ-C: Physical Activity Questionnaire for Older Children.

### Effect of the School-Based Program on AVATAR Modify, Improve, and Maintain Percentile Categories

Descriptive data of health-related quality of life and lifestyle habits in the pre and post conditions expressed in percentage with respect to the modify, improve, and maintain percentile categories are presented in Table 3. After school planning and specific program implementation, the physical well-being dimension in the post condition changed, with an increase in the maintain percentile category and a reduction in the modify and improve percentile categories (Δ: *P*=.001). Psychological well-being perception was augmented in the maintain percentile category, and showed a decrease in the modify percentile category and an enhancement in the improve percentile category (Δ: *P*=.001). The emotion/mood dimension also changed after the school-based program, and this was characterized by a drop in the modify and improve percentile categories in the face of an increase in the maintain percentile category (Δ: *P*=.03). Autonomy, understood as the opportunity to create his/her social and leisure time, improved owing to the specific program, with an increase in the maintain percentile category and a significant decrease in the other percentile categories (Δ: *P*=.001). Moreover, perception of financial resources showed a decrease in the modify percentile category and an increase in the improve percentile category, and demonstrated stability in the maintain percentile category (Δ: *P*=.001). In the social context component, peer relationships showed an increase in the maintain percentile category and a decrease in the modify and improve percentile categories (Δ: *P*=.003). Lastly, the school-based program created according to the precondition results resulted in an improvement in the perception of social acceptance. In fact, for the bullying dimension, there was a significant reduction in the modify percentile category and an increase in the improve percentile category (Δ: *P*=.01).

**Table 3:** Changes in questionnaire findings (pre vs post) according to the modify, improve, and maintain percentile categories.

Variable			Modify percentile category, %					Improve percentile category, %					Maintain percentile category, %					Change (Δ), %							*P* value^a^
			Pre		Post		Pre			Post		Pre			Post		Modify percentile			Improve percentile		Maintain percentile			
**KIDSCREEN-52**																									
	Physical well-being	18.4		18.1		67.4			50.0		14.2			23.9		−0.3			−9.4		9.7		.001		
	Psychological well-being	27.5		7.6		66.8			78.5		5.7			13.9		−19.9			11.8		8.2		.001		
	Mood/emotion	8.8		5.4		78.5			76.1		12.7			18.4		−3.3			−2.4		5.7		.03		
	Self-perception	12.1		10.3		87.9			89.7		0.0			0.0		−1.8			1.8		0.0		.42		
	Autonomy	10.6		4.5		77.3			72.5		12.1			23.0		−6.0			−4.8		10.9		.001		
	Parent relationship	8.8		8.2		91.2			91.8		0.0			0.0		−0.6			0.6		0.0		.89		
	Financial resources	13.3		4.5		86.7			95.5		0.0			0.0		−8.8			8.8		0.0		.001		
	Peers	10.0		6.3		82.2			78.2		7.9			15.4		−3.6			−3.9		7.6		.003		
	School environment	10.3		6.9		77.9			78.2		11.8			14.8		−3.3			0.3		3.0		.12		
	Social acceptance	13.3		7.9		86.7			92.1		0.0			0.0		−5.4			5.4		0.0		.01		
KIDMED^b^			9.4		7.3		76.7			78.2		13.9			14.5		−2.1			1.5		0.6		.44	
PAQ-C^c^			17.8		12.4		80.4			86.4		1.8			3.0		−5.4			4.2		1.2		.07	

^a^*P* value is for the change (Δ).

^b^KIDMED: Mediterranean Diet Quality Index for children and adolescents.

^c^PAQ-C: Physical Activity Questionnaire for Older Children.

## Discussion

### Principal Findings

We explored the development of a web-based school support system for the promotion of health and well-being in adolescents, through a pilot/feasibility study conducted in a small sample of subjects before launching a larger study, thus allowing the definition of public health programs based on the specific needs of the studied population and improving the relationship between schools and research groups.

The AVATAR platform, which processes data automatically and returns the findings legibly and in real time, offers the possibility for schools to have updated and comparable data on adolescents’ behaviors and well-being perceptions to create a network of collaborations with multidisciplinary experts for the development of prevention and health promotion interventions focused on the actual needs detected, which could help in selecting best practices and organizing targeted training sessions.

In addition, according to the technological perspective, the AVATAR platform, which returns the data to the school classified as “modify, improve, and maintain,” is highly flexible and adaptable in its potential applications. This methodological innovation, linked to the innovative approach, involves the simplicity and usability of the data acquired [7]. Teachers, in fact, can directly visualize the classroom and the cluster of students with similar profiles, and analyze a personalized well-being index that provides an integrated and personalized perspective of adolescents’ well-being [6]. As shown by the pilot study, this model helps teachers to more appropriately select interventions and educational programs for individual students and for the classroom, and thus, can monitor compliance and effectiveness. In particular, the data available to the school, in addition to enhancing the school’s success, have preventive purposes and can help improve resilience, happiness, social involvement, self-esteem, and sociability [14,15]. Furthermore, the results obtained by the AVATAR platform allow teachers to measure objectively the perception of students’ health and well-being, and follow their evolution longitudinally in order to reduce risk behaviors or potential risk factors, in line with the primordial prevention statement, which represents one of the aims of the AVATAR platform. The same notion is suitable for promoting social and emotional health in the school context. In fact, the school engaged in the pilot study, in agreement with school health educators who have increased skill development in the dimension of decision-making, had planned specific programs designed to support these areas of learning as closely related to school success and to strengthen relationships with family and peers [21]. This choice, according to the needs that emerged from the first monitoring, is linked to the notion that if adolescents are not conscious of their feelings and emotions, they will find it difficult to make reasoned choices, to choose healthy behaviors, and to achieve a good degree of learning [22].

Health-related quality of life and lifestyle habit data obtained after the school-based program showed better perceptions in the psychological and physical well-being, mood/emotion, self-perception, autonomy, and financial resources dimensions. In the social context, relationships with family and peers, as well as the perception of bullying, improved after didactic actions oriented toward these dimensions. Moreover, in the lifestyle assessment, owing to the school project, adolescents developed higher adherence to a Mediterranean diet and a better physical activity level.

When the data are analyzed according to the AVATAR approach, which involves partitioning into percentiles, what emerges is an improvement in physical and psychological well-being perceptions, emotional responses, and social acceptance with peers, with an increased percentage in the maintain percentile category and a reduction in the modify and improve percentile categories. These results obtained in the pilot study are in line with evidence that considers these variables responsible for healthy behavior and better health-related quality of life [23].

However, despite the objective well-being brought by a specific program aimed to support and potentiate the development of students’ emotional-relational skills and competences, when schools adopt cross-curricular programs, they face different implementation difficulties, such as lack of systematicity and objectivity.

Nevertheless, there are indications, in fact, that school-based programs based on educational activities augment self-image and body satisfaction in adolescents aged 12 to 14 years, probably because the school represents a good platform for increasing empowerment and awareness to health and well-being perceptions [24,25]. According to an ecological model, school-based programs, oriented to increase health status, should consider that school performance not only is the result of learning, but also, above all, depends on the environment in which the student lives and relates, and therefore, on the family microsystem and family-school macrosystem. In this new scenario, the adolescent must become aware of his/her own well-being and have at the same time (by the school) the tools to be proactive and participatory.

To modify individual unhealthy behaviors, it is necessary, therefore, to create environmental conditions suitable for encouraging a healthy lifestyle through an “intersectoral” and transversal approach to risk factors, considering all levels of intervention, both social and psychological. For this, there is a need for more evidence-based school intervention programs and an accurate assessment of their overall efficiency and efficacy.

### Strengths and Limitations

A key strength of the AVATAR platform is that it is the first platform, to our knowledge, designed to introduce a web-based health promotion tool for the school community [26]. Importantly, monitoring the results allows the identification of the strength and fragile characteristics of each adolescent in order to define personalized educational programs. In particular, with the data, the school can (1) carry out longitudinal monitoring of students, (2) orient didactic actions, and (3) promote the personalization of educational and training courses.

In this context, the AVATAR platform intervenes at multiple points in the educational process in young people by combining management and empowerment of health and well-being, applying prevention strategies to reduce disease burden and health expenditure in adulthood, and enhancing learning. The AVATAR platform combines the rigor and objectivity of scientific and technological research with the needs that emerge from the school system regarding health, well-being, and educational success, integrating primordial prevention with the definition of a model of support for the autonomous design of schools. The development of a school-based program from the AVATAR web-based platform represents a powerful pattern for the promotion of health and psychosocial well-being, in which a network of different stakeholders dedicated to education may cooperate together to increase awareness, reduce risk behaviors, and potentiate educational success. Moreover, the AVATAR web-based tool offers a personalized well-being index that may allow the adoption of more individually focused strategies and interventions to improve well-being. Finally, as the AVATAR platform is delivered to students via web-based technology with interactive components through the involvement of teachers, student engagement and program fidelity can be increased, and this is supported by new national and European policies on the welfare of adolescents.

Several limitations should be acknowledged. First, since the questionnaires were completed during a school class, the environment may have biased the students’ responses. Finally, a control group was not included to demonstrate the effectiveness of the targeted intervention based on the needs identified because all schools, due to institutional obligations, carry out projects that could, in any case, impact the sampling results at the end of the school year.

### Conclusion

The AVATAR monitoring platform, as shown by our pilot results, is configured as a tool for enhancing school autonomy, and offers schools and the community the possibility of having updated and comparable data on adolescents’ behaviors and perceptions to create a network of collaborations with multidisciplinary experts for the development of prevention and health promotion interventions focused on the actual needs identified, which could help in selecting best practices and organizing targeted training sessions. Owing to its flexibility and adaptability, the AVATAR platform can be used to (1) promote information campaigns aimed at modifying risk behaviors in the student population, (2) sensitize students and put them at the center of their growth path, (3) inform institutions on the health and well-being of the school population, (4) evaluate the effectiveness of the actions implemented by schools, and (5) promote the exchange of good practices aimed at strengthening systems intended for the education, training, and well-being of students.

## Abbreviations

AVATAR: a new purpose for the promotion and evaluation of health and well-being among healthy teenagers
KIDMED: Mediterranean Diet Quality Index for children and adolescents
PAQ-C: Physical Activity Questionnaire for Older Children

## References

[1] Jones SM, Bouffard SM (2012). Social and Emotional Learning in Schools: From Programs to Strategies and commentaries. Social Policy Report.

[2] Weare K, Nind M (2011). Mental health promotion and problem prevention in schools: what does the evidence say?. Health Promot Int.

[3] Buttitta M, Iliescu C, Rousseau A, Guerrien A (2014). Quality of life in overweight and obese children and adolescents: a literature review. Qual Life Res.

[4] Ross DA, Hinton R, Melles-Brewer M, Engel D, Zeck W, Fagan L, Herat J, Phaladi G, Imbago-Jácome D, Anyona P, Sanchez A, Damji N, Terki F, Baltag V, Patton G, Silverman A, Fogstad H, Banerjee A, Mohan A (2020). Adolescent Well-Being: A Definition and Conceptual Framework. J Adolesc Health.

[5] Ravens-Sieberer U, Herdman M, Devine J, Otto C, Bullinger M, Rose M, Klasen F (2014). The European KIDSCREEN approach to measure quality of life and well-being in children: development, current application, and future advances. Qual Life Res.

[6] Mastorci F, Bastiani L, Doveri C, Trivellini G, Casu A, Vassalle C, Pingitore A (2020). Adolescent Health: A Framework for Developing an Innovative Personalized Well-Being Index. Front Pediatr.

[7] Trivellini G, Doveri C, Mastorci F, Bastiani L, Cappa C, Vassalle C, Pingitore A (2018). Innovative web-based tool for promoting well-being among healthy adolescents: An implementation protocol. J Transl Sci.

[8] de Onis M, Onyango AW, Borghi E, Siyam A, Nishida C, Siekmann J (2007). Development of a WHO growth reference for school-aged children and adolescents. Bull World Health Organ.

[9] Ravens-Sieberer U, Gosch A, Rajmil L, Erhart M, Bruil J, Duer W, Auquier P, Power M, Abel T, Czemy L, Mazur J, Czimbalmos A, Tountas Y, Hagquist C, Kilroe J, European Kidscreen Group (2005). KIDSCREEN-52 quality-of-life measure for children and adolescents. Expert Rev Pharmacoecon Outcomes Res.

[10] The KIDSCREEN Group Europe (2006). The KIDSCREEN Questionnaires - Quality of life questionnaires for children and adolescents.

[11] Berra S, Ravens-Sieberer U, Erhart M, Tebé C, Bisegger C, Duer W, von Rueden U, Herdman M, Alonso J, Rajmil L, European KIDSCREEN group [kidscreen] (2007). Methods and representativeness of a European survey in children and adolescents: the KIDSCREEN study. BMC Public Health.

[12] Serra-Majem L, Ribas L, Ngo J, Ortega RM, García A, Pérez-Rodrigo C, Aranceta J (2004). Food, youth and the Mediterranean diet in Spain. Development of KIDMED, Mediterranean Diet Quality Index in children and adolescents. Public Health Nutr.

[13] Saint-Maurice PF, Welk GJ, Beyler NK, Bartee RT, Heelan KA (2014). Calibration of self-report tools for physical activity research: the Physical Activity Questionnaire (PAQ). BMC Public Health.

[14] Mastorci F, Piaggi P, Bastiani L, Trivellini G, Doveri C, Casu A, Vassalle C, Pingitore A (2020). The impact of menarche on health-related quality of life in a sample of Italian adolescents: evidence from school-based AVATAR project. Eur J Pediatr.

[15] Mastorci F, Bastiani L, Trivellini G, Doveri C, Vassalle C, Pingitore A (2020). A new integrated approach for adolescent health and well-being: the AVATAR project. Health Qual Life Outcomes.

[16] Patton GC, Coffey C, Cappa C, Currie D, Riley L, Gore F, Degenhardt L, Richardson D, Astone N, Sangowawa AO, Mokdad A, Ferguson J (2012). Health of the world's adolescents: a synthesis of internationally comparable data. The Lancet.

[17] Patton GC, Sawyer SM, Santelli JS, Ross DA, Afifi R, Allen NB, Arora M, Azzopardi P, Baldwin W, Bonell C, Kakuma R, Kennedy E, Mahon J, McGovern T, Mokdad AH, Patel V, Petroni S, Reavley N, Taiwo K, Waldfogel J, Wickremarathne D, Barroso C, Bhutta Z, Fatusi AO, Mattoo A, Diers J, Fang J, Ferguson J, Ssewamala F, Viner RM (2016). Our future: a Lancet commission on adolescent health and wellbeing. The Lancet.

[18] Lamblin M, Murawski C, Whittle S, Fornito A (2017). Social connectedness, mental health and the adolescent brain. Neurosci Biobehav Rev.

[19] Rajmil L, Alonso J, Berra S, Ravens-Sieberer U, Gosch A, Simeoni M, Auquier P, KIDSCREEN group (2006). Use of a children questionnaire of health-related quality of life (KIDSCREEN) as a measure of needs for health care services. J Adolesc Health.

[20] Berman AH, Liu B, Ullman S, Jadbäck I, Engström K (2016). Children's Quality of Life Based on the KIDSCREEN-27: Child Self-Report, Parent Ratings and Child-Parent Agreement in a Swedish Random Population Sample. PLoS One.

[21] Elias M, Weissberg R (2000). Primary prevention: educational approaches to enhance social and emotional learning. J Sch Health.

[22] Perrin JM, Duncan G, Diaz A, Kelleher K (2020). Principles And Policies To Strengthen Child And Adolescent Health And Well-Being. Health Aff (Millwood).

[23] Weissberg RP, Elias MJ (1993). Enhancing young people's social competence and health behavior: An important challenge for educators, scientists, policymakers, and funders. Applied and Preventive Psychology.

[24] Stice E, Becker CB, Yokum S (2013). Eating disorder prevention: current evidence-base and future directions. Int J Eat Disord.

[25] Xu T, Tomokawa S, Gregorio ER, Mannava P, Nagai M, Sobel H (2020). School-based interventions to promote adolescent health: A systematic review in low- and middle-income countries of WHO Western Pacific Region. PLoS One.

[26] Langford R, Bonell C, Jones H, Pouliou T, Murphy S, Waters E, Komro K, Gibbs L, Magnus D, Campbell R (2015). The World Health Organization's Health Promoting Schools framework: a Cochrane systematic review and meta-analysis. BMC Public Health.

